# Chronic traumatic encephalopathy in two former Australian National Rugby League players

**DOI:** 10.1186/s40478-019-0751-1

**Published:** 2019-06-27

**Authors:** Michael E. Buckland, Joanne Sy, Istvan Szentmariay, Alexandra Kullen, Maggie Lee, Antony Harding, Glenda Halliday, Catherine M. Suter

**Affiliations:** 1 Department of Neuropathology, Royal Prince Alfred Hospital, 94 Mallet St, Camperdown, NSW 2050 Australia; 2 Discipline of Pathology, School of Medical Sciences, Brain & Mind Centre, University of Sydney, Camperdown, NSW 2006 Australia; 3 Forensic and Analytical Science Service, Lidcombe, NSW 2141 Australia; 4 Central Clinical School, Brain & Mind Centre, University of Sydney, Camperdown, NSW 2006 Australia; 5 Faculty of Medicine, University of New South Wales, Kensington, NSW 2052 Australia

**Keywords:** Chronic traumatic encephalopathy, CTE, Rugby league, Tau, ARTAG

To the Editor:

Historically the term chronic traumatic encephalopathy (CTE) was used synonymously with ‘punch-drunk’ to describe the neurological deficits of ex-boxers thought due to repeated blows to the head [3]. In the twenty-first century, CTE has come to define a neuropathological diagnosis associated with repeated head injury, with the entity-defining lesion consisting of phosphorylated Tau (pTau) accumulation in neurons and astrocytes in a perivascular distribution at the depths of sulci [8]. The clinical presentation of CTE bears many features similar to the punch-drunk syndrome as originally described [2, 12]. A recent retrospective analysis of the brains in the seminal 1973 Corsellis study of punch-drunk ex-boxers boxers found that half (7 of 14) had neuropathology definitive of CTE [6].

While the above would appear to provide persuasive evidence in support of a distinct disease associated with repetitive head injury in sport, there remains a fair degree of scepticism over CTE (e.g [11]). This is due in large part to the changing semantics of the term, and to the lack of a distinct or discrete clinical syndrome. In the USA, active research into CTE in the National Football League is going some way to address the latter issue, but in other sporting nations such as Australia, awareness of the potential for CTE in contact sports is hampered by the absence of clear cases in the literature. Here we report the finding of CTE pathology in the brains of two former Australian National Rugby League (NRL) players. To our knowledge these are the first reported cases of CTE in rugby league in the world, and only the second and third cases of CTE ever reported in Australian sportspeople.

Both cases were middle-aged ex-professionals who had each played more than 150 first grade NRL games over many years. Case1 had a successful career after retirement, and had been working up until his death. He did not abuse tobacco, alcohol, or other drugs. Family members reported increasing reliance on aide-mémoires for daily activities in the years prior to death, and recent difficulties remembering details of a significant life event. Case2 had some issues during his transition to a post-playing career, but was productively employed up until his death.

Table 1 summarises the pertinent neuropathology of each case. Macroscopically, neither exhibited brain atrophy or evidence of prior traumatic brain injury. Case1 had a small cavum septum pellucidum. Microscopically, both cases had neocortical foci of the pathognomonic lesions of CTE: pTau (AT8) accumulation concentrated irregularly at the sulcal depths in a perivascular distribution. pTau was present in neurons as tangle and pretangle pathology, as well as in neurites and astrocytes. In Case1, eight definitive CTE foci were found across six of eight neocortical blocks examined (bilateral superior/middle frontal gyri, superior/middle temporal gyri, and superior parietal lobules) (Fig. 1a, b); pTau was absent in occipital lobes (striate and peristriate cortex). In Case2, four CTE foci were found in two of four neocortical blocks examined (superior/middle frontal gyri, superior/middle temporal gyri) (Fig. 1c, d). In non-sulcal neocortex, neurons with tangle and pretangle pathology were present mostly in cortical layers II and III (moderate frequency in Case2, low in Case1).

**Table 1:** Summary of neuropathogical features of brains of two former NRL players

	Case1	Case2
Tau pathology		
Depths of cortical sulci (neuronal & astrocytic), perivascular	Present	Present
Superficial cortical layers	Present	Present
CA2 (neuronal, neuritic), CA4 (proximal dendritic swellings)	Present	Absent
Substantia nigra and raphe nuclei (neuronal, neuritic)	Present	Present
Subpial & periventricular thorny astrocytes	Present	Present
Large grain-like & dot-like structures	Present	Present
Other supportive pathology		
Dilatation of IIIrd ventricle	Absent	Absent
Septal abnormalities	Present	Absent
Other pathology		
Vascular disease	Absent	Absent
Beta-A4 (amyloid)	Present (Thal phase 1)	Absent
CERAD score	0	0
pTDP-43	Present (*amygdala, hippocampus, entorhinal cortex, inferior temporal cortex)*	Absent
Alpha-synuclein	Absent	Absent
ARTAG	moderate (medial temporal)	mild (medial temporal)
Diagnosis	Stage 3 CTE	Stage 2 CTE

**Fig. 1:**
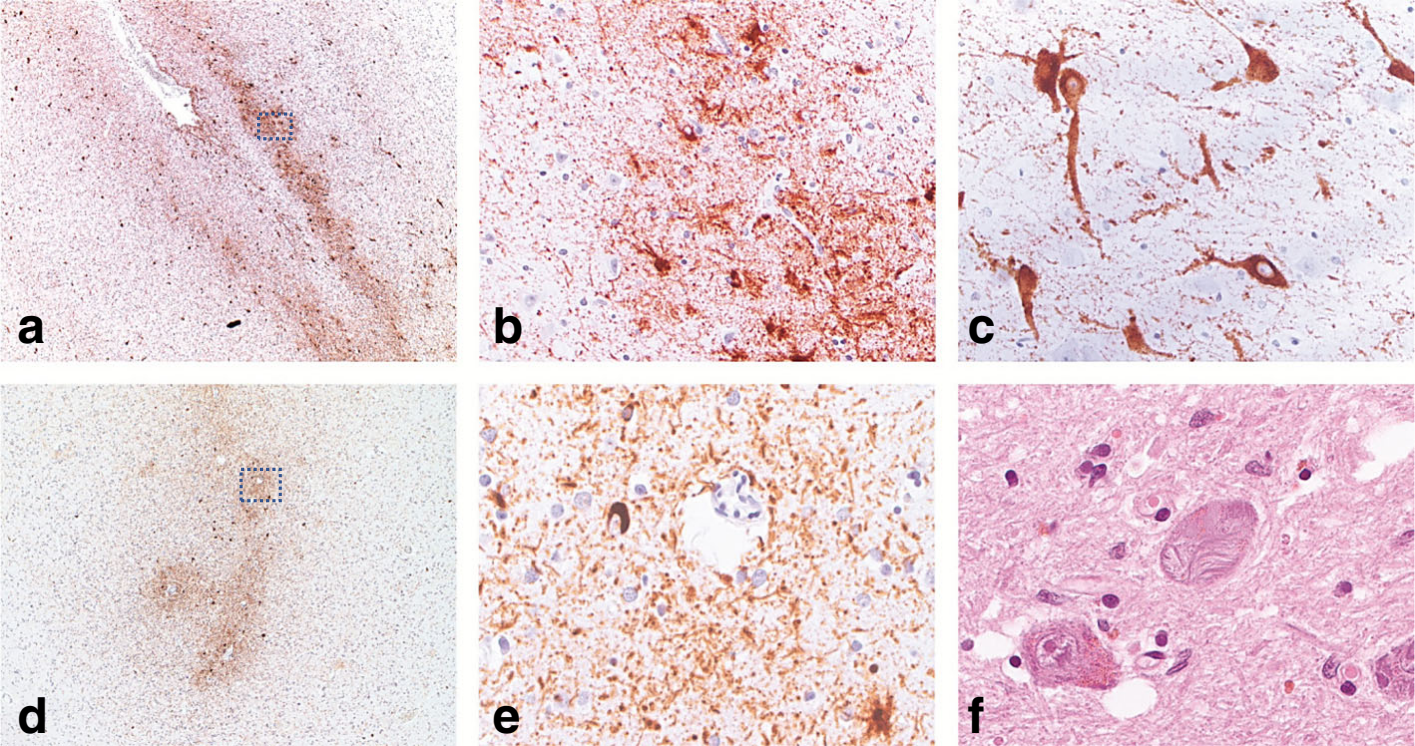
Phosphorylated Tau immunohistochemistry (**a**-**e**) and haematoxylin and eosin staining (**f**) in Case1 (**a**-**c**) and Case2 (**d**-**f**). (**a**) pTAu staining is irregularly distributed at the sulcal depths in both neurons and glia. (**b**) high power view of boxed area in (**a**) showing perivascular accentuation of pTau staining. (**c**) prominent proximal dendrite staining with pTau in CA4 neurons in Case1. (**d**) perivascular pTAu staining in sulcal depths of Case2 (frontal cortex). (**e**) high power view of boxed area in (**d**). (**f**) globose fibrillary tangles in the substantia nigra with some pigment incontinence and gliosis in Case2

Case1 also demonstrated medial temporal lobe involvement, with neuronal loss in the hippocampal CA1 region, subiculum, and amygdala, with prominent ghost tangle formation in amygdala. pTau staining was widespread, with notable pretangle neuronal staining in CA4 and CA2 regions, with prominent staining of proximal dendritic segments in CA4 (Fig. 1e). Both cases had some Tau-positive globose neurofibrillary tangles (GFTs; Fig. 1f) and neurites in the substantia nigra, accompanied by mild neuronal loss, gliosis, and pigment incontinence. Case1 also showed involvement of raphe nuclei, locus coeruleus and dorsal motor nucleus of vagus. Thorny astrocytic Tau staining in subpial and subependymal locations in medial temporal lobe was also seen in both cases, indicating co-existent aging-related tau astrogliopathy (ARTAG) [7].

In both cases, the neuronal inclusions comprised both 3R and 4R Tau isoforms, with 4R Tau preferentially labelling neuritic and glial pathology. In the ghost tangles of Case 1, 3R Tau was the dominant isoform. Case 1 also contained medial temporal lobe TDP-43 pathology, and low numbers of diffuse plaques in frontal and temporal neocortex. Coexistent pathology is detailed in Table 1. The distinctive Tau pathology seen in both cases is diagnostic of CTE; the medial temporal lobe involvement indicates that Case1 corresponds to stage III pathology, while Case2 best corresponds to Stage II pathology [9].

Rugby league is a very popular amateur and professional sport in Australia with a player base of around half a million people https://www.clearinghouseforsport.gov.au/. It is distinct from rugby union (‘rugby’), having established itself as a separate sporting code over 100 years ago. It is also popular in Pacific Island nations, New Zealand, the United Kingdom and France. There is frequent high force tackling of players, and head impact during tackling is common. The rate of concussions in professional rugby league is estimated at 8.92 per 1000 match hours (i.e. one concussion every 3.35 games [4]), and may be significantly higher in youth rugby league [5]. Currently, repeated mild traumatic brain injury, both concussive and subconcussive, is the only known likely risk factor for CTE [1]. There is little available data on long-term neurological outcomes in rugby league players, however a recent assessment of 25 retired NRL players identified significant motor and cognitive changes, along with neurophysiological alterations compared with matched controls with no history of contact sports [10].

While this report is limited by the paucity of antemortem clinical information, the finding of two clear CTE diagnoses in our department is nonetheless remarkable. RPA Neuropathology is the only neuropathology department in the most populous state in Australia. Over the last four years, the routine service has examined 470 brain autopsy specimens, with 50 undergoing neocortical Tau immunostaining for various indications. These cases we present here are the only two cases of CTE we have identified in routine practice, and it is noteworthy that they are both in elite rugby league players.

Given that brain autopsy is rarely pursued these days, even in cases of suicide referred to the Coroner, it is difficult to assess whether these two CTE cases are serendipitous findings, or emblematic of a more common issue with Rugby League and other Australian football codes. Our findings underscore the urgent need for further studies into CTE in sportspeople and other at-risk populations internationally, in order to define and act upon the occupational and public health implications of this disease.

## Data Availability

Data sharing is not applicable to this article (no datasets were generated or analysed during the current study).

## Abbreviations

ARTAG: Aging-related tau astrogliopathy
CTE: Chronic traumatic encephalopathy
NRL: National rugby league
