# Correction to: Chronic traumatic encephalopathy in two former Australian National Rugby League players

**DOI:** 10.1186/s40478-019-0772-9

**Published:** 2019-07-29

**Authors:** Michael E. Buckland, Joanne Sy, Istvan Szentmariay, Alexandra Kullen, Maggie Lee, Antony Harding, Glenda Halliday, Catherine M. Suter

**Affiliations:** 1 Department of Neuropathology, Royal Prince Alfred Hospital, 94 Mallet St, Camperdown, NSW 2050 Australia; 2 Discipline of Pathology, School of Medical Sciences, Brain & Mind Centre, University of Sydney, Camperdown, NSW 2006 Australia; 3 Forensic and Analytical Science Service, Lidcombe, NSW 2141 Australia; 4 Central Clinical School, Brain & Mind Centre, University of Sydney, Camperdown, NSW 2006 Australia; 5 Faculty of Medicine, University of New South Wales, Kensington, NSW 2052 Australia

**Correction to: Acta neuropathol commun (2019) 7: 97.**

**https://doi.org/10.1186/s40478-019-0751-1**

In the original publication of this article [1] the term ‘National Rugby League (NRL)’ was used to refer to professional rugby league competition sport in Australia. The term should have read ‘professional rugby league’ to include the various professional competition nomenclatures over the last fifty years, including but not limited to NRL. In this correction article, the incorrect and correct information are published.

Due to these changes the term ‘NRL’ should be disregarded from the abbreviation list.

**Table:** 

Incorrect	Corrected information
Chronic traumatic encephalopathy in two former Australian **National Rugby League** players	Chronic traumatic encephalopathy in two former Australian **professional rugby league** players
Here we report the finding of CTE pathology in the brains of two former Australian **National** Rugby League (NRL) players.	Here we report the finding of CTE pathology in the brains of two former Australian **professional** rugby league players.
Both cases were middle-aged ex-professionals who had each played more than 150 first grade **NRL** games over many years.	Both cases were middle-aged ex-professionals who had each played more than 150 first grade **professional rugby league** games over many years.
There is little available data on long-term neurological outcomes in rugby league players, however a recent assessment of 25 retired **NRL** players identified significant motor and cognitive changes, along with neurophysiological alterations compared with matched controls with no history of contact sports [13].	There is little available data on long-term neurological outcomes in rugby league players, however a recent assessment of 25 retired **professional rugby league** players identified significant motor and cognitive changes, along with neurophysiological alterations compared with matched controls with no history of contact sports [13].
Table 1. Summary of neuropathogical features of brains of two former **NRL** players	Table 1. Summary of neuropathogical features of brains of two former **professional rugby league** players
